# Structural Basis of Intermolecular Interactions Between APOBEC3 and HIV-1 Vif

**DOI:** 10.3390/v18070787

**Published:** 2026-07-19

**Authors:** Hirotaka Ode, Yasumasa Iwatani

**Affiliations:** 1 Department of Infectious Diseases and Immunology, Clinical Research Center, NHO Nagoya Medical Center, Nagoya 460-0001, Aichi, Japan; ode.hirotaka.pw@mail.hosp.go.jp; 2 Department of Microbiology & Immunology, Hamamatsu University School of Medicine, Hamamatu 431-3192, Shizuoka, Japan

**Keywords:** APOBEC3, Vif, E3 ubiquitin ligase, host restriction factor, CBF-β, structure, cryo-EM, interface, RNA

## Abstract

The human APOBEC3 (A3) family of cytidine deaminases, including A3G, A3F, and A3H, participates in cellular anti-retroviral immunity. In contrast, to antagonize the anti-retroviral activities of these A3 family proteins, HIV-1 produces its gene product called viral infectivity factor (Vif) in infected cells. Vif is a pleiotropic hub protein that specifically binds to various A3 proteins with the aid of host core-binding factor subunit β (CBF-β) and mediates their proteasomal degradation. To date, numerous biological and structural studies have been performed to understand the arms race between A3 and Vif. Previous extensive mutagenesis and structural analyses have suggested that there are three distinct types of Vif-binding interfaces among human A3s and three largely nonoverlapping interfaces on Vif for binding with these A3s. Moreover, recent cryo-electron microscopy (cryo-EM) structural analyses have clarified further details of the different intermolecular interactions of Vif with each of three human A3s (A3G, A3F, and A3H) and have proposed a possible mechanism by which one Vif molecule can recognize all three types of A3s. In this review, we summarize the current understanding of the structural basis of the interaction between A3 and Vif. This information may be helpful for developing drugs targeting these interfaces.

## 1. Introduction

Apolipoprotein B mRNA-editing enzyme, catalytic polypeptide-like 3 (APOBEC3, hereafter A3) proteins are a family of cytidine deaminases that restrict viral replication in eutherian mammals [1,2]. In the case of primates, the genomes encode seven A3 family proteins (A3A, A3B, A3C, A3D, A3F, A3G, and A3H) [3,4,5]. Moreover, polymorphic variants are found in some of these genes, as exemplified by human A3H, which has at least 12 different haplotypes [6,7,8,9,10]. From a structural perspective, each A3 has one or two phylogenetically related cytidine deaminase domains. A3A, A3C, and A3H consist of single domains, whereas A3B, A3D, A3F, and A3G are composed of N-terminal and C-terminal domains (NTD and CTD, respectively). Human A3G, A3F, and A3H haplotype II (hapII) are potent restriction factors against human immunodeficiency virus type 1 (HIV-1), other lentiviruses, and endogenous retroelements (reviewed in [11,12,13]). These three A3 family proteins have the potential to produce defective HIV-1 variants through the introduction of G-to-A hypermutation, which is dependent on their cytidine deaminase activities, while they can also bind the HIV-1 RNA genome to block its reverse transcription in a deaminase-independent manner. In the natural target cells of HIV-1, including human primary CD4^+^ T cells and macrophages, interferon-associated immune responses upregulate the expression levels of these proteins [14].

**Figure 1:**
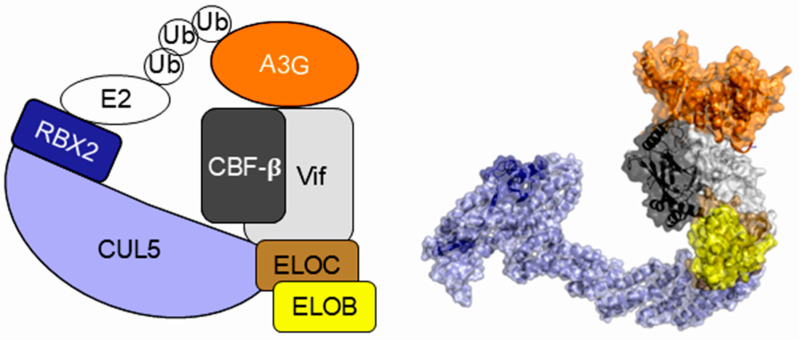
A schematic diagram and model structure of the Vif-mediated E3 ubiquitin (Ub) ligase complex bound to APOBEC3G (A3G) with multiple ubiquitins conjugated (**left**). The structure excluding E2 was constructed by superimposing three complex structures (PDB IDs 8CX0, 4N9F, and 6V9I) using PyMOL (Schrödinger) and is displayed with solid ribbons and transparent surfaces (**right**). CUL5: Cullin-5, ELOB: Elongin B, ELOC: Elongin C, RBX2: RING-box protein 2.

To antagonize the restriction by A3 proteins and produce nascent infectious viral particles, lentiviral genomes encode a viral accessory protein called viral infectivity factor (Vif). In primate lentiviruses such as HIV-1 and simian immunodeficiency virus (SIV), Vif plays a role as a pleiotropic hub protein that binds to various A3 proteins with host transcription co-factor CBF-β scaffolding and hijacks a cellular Cullin-5 (CUL5)-E3 ubiquitin ligase complex to promote the degradation of A3s [15,16,17] (Figure 1). The *vif* gene is thought to have evolved to adapt to the A3 repertoire of a new host after every cross-species transmission event of an ancestral SIV [18,19]. As a consequence of this arms race between the virus and its host, current HIV-1 Vif variants can counteract a repertoire of human A3 family proteins efficiently and specifically.

To date, cumulative mutagenesis studies have identified residues responsible for the binding of HIV-1 Vif to human A3G, A3F, and A3H hapII. In the double-domain proteins A3G and A3F, these responsible residues are localized in one of two domains (the A3G NTD and A3F CTD). Mapping of these responsible residues onto the crystal structures of the Vif and A3 domains has further revealed the three-dimensional positions of the binding interfaces on them [20,21,22]. Moreover, recent advances in cryo-electron microscopy (cryo-EM) structural analysis technology have enabled the structural determination of large complex molecules, including HIV-1 Vif bound to the A3 family protein in the presence or absence of components of E3 ubiquitin ligase (e.g., CUL5, EloB, and EloC) [23,24,25,26,27,28]. A series of these structures of the A3-Vif complexes has provided much clearer insights into A3-Vif interactions. Here, we summarize the current understanding of the A3-Vif interaction from a structural perspective.

## 2. Presumed A3-Vif Interfaces Based on Extensive Mutagenesis

**Figure 2:**
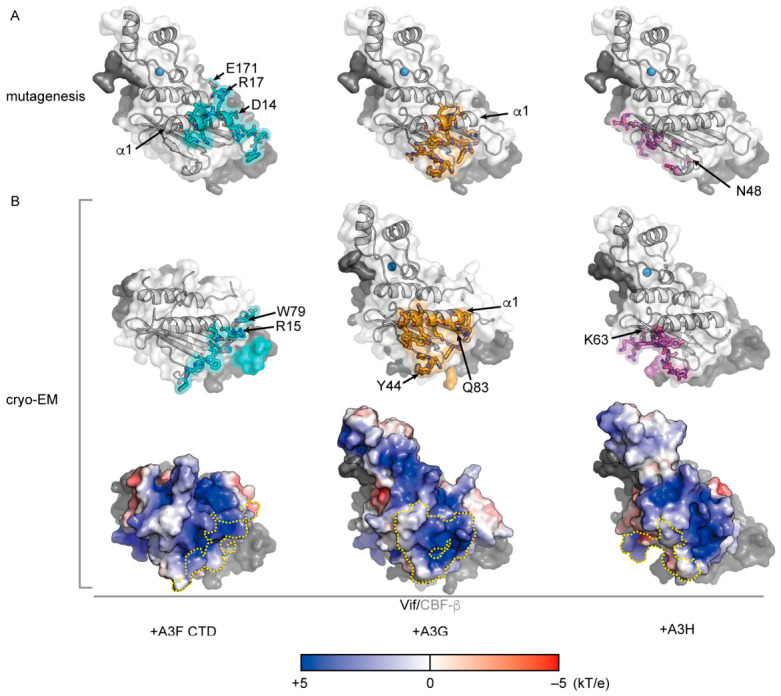
A3-binding interfaces on Vif. (**A**) The crystal structure of the Vif-CBF-β complex (PDB 4N9F) is shown to map the residues responsible for A3-Vif interactions proposed by mutagenesis studies, although there is no information on the Vif C-terminal residues (D172, R173, W174) crucial for A3F binding. The responsible residues are highlighted with sticks colored in cyan (A3F-Vif), light orange (A3G-Vif), and violet (A3H-Vif). Vif is depicted with ribbons and a semitransparent surface, whereas CBF-β is represented by a dark gray surface. The zinc ion is shown as an aqua sphere. (**B**) The cryo-EM structures of the Vif-CBF-β components in the complexes (PDB 6NIL, 8CX0, and 8FVI for Vif bound to the A3F CTD, A3G, and A3H, respectively) are shown to highlight the Vif residues within 4 Å of the A3-Vif interfaces. Electrostatic potentials on the Vif surfaces based on the cryo-EM structures are also shown using a blue–white–red gradient color scale ranging from +5 (blue) to −5 kT/e (red) (the bottom color bar). The binding interfaces are enclosed by yellow dotted lines. The structures were drawn with PyMOL v2. The important Vif residues (D14, R15, R17, Y44, N48, K63, W79, Q83, E171) and helix α1 are highlighted by using allows.

**Figure 3:**
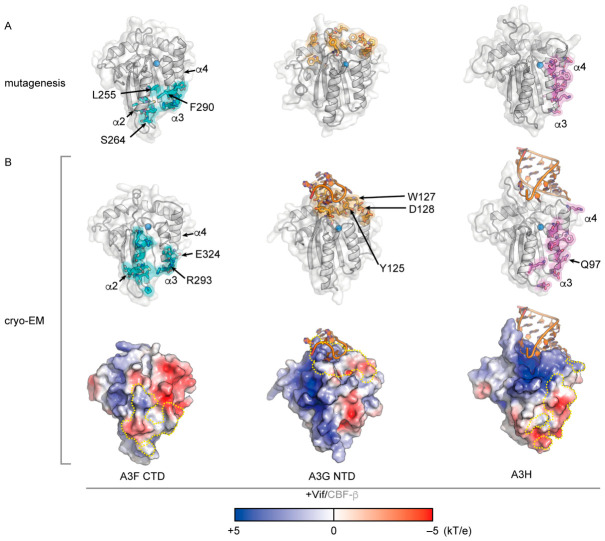
Vif-binding interfaces on A3. (**A**) Structures of the Vif-binding domains in each A3 (PDB 3WUS for the human A3F CTD, a model structure based on PDB 5K81 for the human A3G NTD, and PDB 6B0B for the human A3H hapII protomer) are shown to map the residues responsible for the A3-Vif interactions proposed by mutagenesis studies. These responsible residues are highlighted in a similar manner to those in Figure 2 (cyan for A3F, light orange for A3G, and violet for A3H). Each A3 is depicted with ribbons and a semitransparent surface. RNA and a zinc ion are represented by an orange ribbon and an aqua sphere, respectively. (**B**) The cryo-EM structures of the Vif-binding A3 domains in the complexes (PDB 6NIL for A3F CTD, 8CX0 for A3G, and 8FVI for A3H) are shown to highlight the A3 residues within 4 Å of the A3-Vif interfaces. Electrostatic potentials on the A3 surfaces based on the cryo-EM structures are also displayed using a blue–white–red gradient color scale ranging from +5 (blue) to −5 kT/e (red), the same color scale as that in Figure 2 (the bottom color bar). The binding interfaces are enclosed by yellow dotted lines. All the structures were drawn with PyMOL v2. The important residues (L255, S264, F290, R293, E324 in A3F CTD, Y125, W127, D128 in A3G NTD, Q97 in A3H) and helices (A3F CTD α2, α3 and α4, A3H α3 and α4) are highlighted by using allows.

Previous extensive mutagenesis and crystal structure analyses have provided valuable insights into the positions of A3-binding interfaces on Vif and their corresponding Vif-binding interfaces on each A3 domain. When residues crucial for A3-Vif binding were mapped in the reported structures of the Vif [29] (PDB 4N9F) and the A3 domains [13,22], the A3-Vif interfaces could be identified. Importantly, three largely nonoverlapping interfaces on Vif have been suggested for binding to A3G, A3F, and A3H (Figure 2A) [20]. Conversely, according to a model structure of human A3G NTD [21] based on the crystal structure of rhesus A3G NTD [30] (PDB 5K81) and the crystal structures of human A3F CTD [31] (PDB 3WUS) and human A3H [32] (PDB 6B0B), these A3 domains likely have three distinct types of HIV-1 Vif-binding interfaces on similar structural folds [21,22] (Figure 3A). It has also been reported that human A3C and A3D, but not A3A and A3B, have Vif-binding capacity and can interact with Vif in a manner similar to that of human A3F because of their high sequence identities [31,33]. Taken together, it is assumed that the Vif-binding interfaces among the human A3 family fall into three distinct types: A3G, A3C/D/F, and A3H, and that Vif possesses the corresponding three different A3-binding interfaces.

## 3. A3F-Vif Interactions

Recently, cryo-EM structural studies have determined a series of complex structures of Vif with A3G, A3F CTD, and A3H and have clarified the molecular details of A3-Vif interactions. The first reported structure of the A3-Vif complex is the cryo-EM structure of the trimeric complex of the A3F CTD, Vif, and CBF-β [23] (PDB 6NIL). This structure was solved by using a fusion protein of a human A3F CTD mutant with solubility-enhancing mutations linked to CBF-β via a 40-amino-acid linker to stabilize the complex structure with Vif. Structural data revealed that the downstream flanking loop of the A3F CTD α2 helix has direct contact with hydrophobic Vif residues (W79 and L81 in the loop between β4 and β5) and a charged residue (R15 in the N-terminal edge of the α1 helix, also known as the ^14^DRMR^17^ motif). In addition, a salt bridge is formed between the charged residue A3F E289 in the α2 helix and K50 in the upstream flanking loop of β3 of Vif (Figure 2B and Figure 3B). The structure also indicated additional characteristics, i.e., direct interaction of the A3F CTD with CBF-β. Salt bridges are formed between charged residues in the A3F CTD α3/α4 helices (R293 and E324) and CBF-β (E54 and R35/R43).

However, the complex structure information only partially explains the previously proposed A3F-Vif interface that was identified via extensive structure-guided mutagenesis [31,33,34,35,36] (Figure 2 and Figure 3). The previously proposed interface additionally includes A3F residues within a shallow cavity between the α2 and α3 helices (L255, S264, and F290), as well as Vif residues around the α1 helix edge (D14, M16, and R17) and in discontinuous flexible loops (E74 and E171-W174). Moreover, reciprocal mutagenesis analysis in a previous biochemical study has suggested that Vif R15 interacts with A3F E289 [36], which is contradictory with the observation from the cryo-EM structure. Therefore, further validation of the A3F-Vif interaction remains necessary.

## 4. A3G-Vif Interactions

Several cryo-EM structures of A3G-Vif complexes have been reported [24,25,26]. Gross et al. succeeded in determining the structure of human A3G in complex with HIV-1 Vif, CBF-β, EloB, and EloC [24] (PDB 8CX0). Notably, the structure revealed that the complex additionally contains a purine-rich single-stranded RNA (ssRNA) sandwiched between A3G and Vif, which appears to function as a molecular glue. The ssRNA binds to positively charged patches on the A3G and Vif surfaces [29,37]. Two flanking purine bases of the ssRNA are buried in a pocket formed by discontinuous loops of the human A3G NTD (I26, W94, Y124, Y125, and W127) and a Vif 3_10_ helix (H42, H43, and Y44 in the well-known ^40^YRHHY^44^ motif), while the phosphate backbone of the ssRNA forms hydrogen bonds and salt bridges with A3G (Y124 and Y125) and Vif (K22/K26 on α1 and Y40 in the ^40^YRHHY^44^ motif). On the basis of hydrogen bonding patterns, adenine may be preferable for the two ssRNA bases. Moreover, the A3G ^128^DPD^130^ motif interacts directly with Vif (R15 and Q83 at the edges of the α1 helix and β5 strand, respectively) via salt bridges and hydrogen bonds. Most of the amino acid residues at the A3G-ssRNA-Vif interface were consistent with those in previously determined mutagenesis studies for the importance of the interaction [3,13,20] (Figure 2B and Figure 3B). For example, the A3G D128 and K128 residues, which naturally occur in hominoids and Old World monkeys (OWMs), respectively [38], are critical for phenotypes sensitive or resistant to HIV-1 Vif-mediated degradation [39,40,41]. In contrast, Vif variants of HIV-1 and SIV infected in chimpanzees can adapt to A3G D128 by bearing Q83 or H83, whereas it is likely that the presence of Tyr (Y) at the corresponding position of SIV Vif adapted in OWMs is suitable for A3G K128 [42]. Taken together, these complex structures clarify that HIV-1 Vif interacts with A3G not only directly but also through ssRNA as an intermediary. Although human A3G self-associates upon its packaging into nascent viral particles [43], the interfaces of human A3G for Vif binding and for self-association are mutually exclusive [24].

This architecture of the A3G-ssRNA-Vif interaction is consistently found in the other two cryo-EM structures of HIV-1 Vif in complex with either a solubility-enhanced human A3G mutant [25] (PDB 8H0I) or an HIV-1 Vif-sensitive OWM A3G mutant bearing K128D [26] (PDB 8E40). The root-mean-square deviations (RMSDs) of the main chain atoms (N, Cα, and C) for all pairwise comparisons among the three structures, estimated by the “align” command in PyMOL v2 (Schrödinger), are less than 2.3 Å, underscoring the structural similarity among the three structures and the structural relevance of the A3G-ssRNA-HIV-1 Vif interaction.

The involvement of ssRNA in the A3G-Vif interaction is most likely due to A3G’s strong binding capacity to ssRNA, with a preference for purine bases [44,45,46]. Notably, extremely high proportions of adenine are characteristic of lentiviral RNA genomes [47,48,49]. This speculation is further supported by the crystal structure of rhesus A3G bound predominantly to ssRNA (PDB 7UU4), which suggests that even in the absence of Vif, A3G can form consistent interactions with two key adenines in an ssRNA molecule [46]. Hence, this evidence suggests that Vif recognizes the ssRNA-binding state of A3G rather than recruiting ssRNA to the A3G-Vif complex, although Vif is an RNA-binding protein as well [50,51,52,53].

## 5. A3H-Vif Interactions

Initially, the determination of three X-ray crystal structures of pig-tail macaque, chimpanzee and human hapII A3Hs revealed that these primate A3Hs commonly form homodimers bridged by a short double-stranded RNA (dsRNA) [32,54,55]. Chimpanzee A3H (cpzA3H), which exhibits higher solubility than other primate A3Hs, is more sensitive to HIV-1 Vif than human A3H hapII due to an amino acid substitution at position 97 (Q97 in chimpanzee versus K97 in humans) [21]. Based on the cpzA3H homodimer structure, the putative Vif-interaction interface in the A3H dimer was predicted to reside within a single A3H unit, remaining accessible to Vif without potential steric hindrances, despite the dsRNA-mediated dimerization. Therefore, it was hypothesized that two units of the Vif– CBF-β complex could bind simultaneously to one A3H dimer.

Recently, our group and others resolved the complex structures of A3H with HIV-1 Vif, CBF-β, CUL5, ELOB, and ELOC [27,28] (PDB 8FVI and 9E93), revealing a consistent architecture of A3H-Vif interactions. Unexpectedly, within the stable dsRNA-mediated homodimer, Vif directly binds with only one of the two A3H protomers (the proximal A3H). In these complex structures, the dsRNA remains bound to the proximal A3H protomer, whereas there is no obvious ssRNA-mediated interaction between A3H and Vif unlike the A3G-Vif complex [24,25,26]. Notably, the structure of the other A3H protomer (the distal A3H) was poorly determined, likely due to occasional dissociation or high flexibility. These observations might explain why only the proximal A3H protomer is selectively disassembled from the stable dimer for subsequent proteasomal degradation [28].

The intermolecular interactions between A3H and Vif are constituted primarily by the α3 and α4 helices in A3H and the β-sheet (β2–β6 strands) in HIV-1 Vif, which is in agreement with previous reports based on mutagenesis experiments [21,56] (Figure 2 and Figure 3). Intriguingly, the A3H Q97 residue forms intermolecular hydrogen bonds with Vif K63. Therefore, the K97 residue in wild-type human A3H hapII reduces Vif susceptibility.

However, even after the complex structures have been determined, it remains unclear how the Vif H48 residue facilitates the degradation of human A3H hapII but not Vif N48 [57]. This is because the Vif residue at position 48 is distal from the A3H interface, according to the determined structures. It is assumed that this residue could allosterically regulate the interactions of A3H with Vif and/or CBF-β [27].

Additionally, unstable human A3H variants with a weak dsRNA-binding capacity, such as human A3H hapI, undergo proteasomal degradation after they are translocated to the nucleus even in the absence of Vif [55,58]. A recent study has revealed that this degradation is mediated primarily by distinct ubiquitin E3 ligases (UBR4, UBR5, and HUWE1) [59], although how the components of these ligase complexes interact with A3H remains elusive.

## 6. Similarities and Differences in A3-Vif Interactions

**Figure 4:**
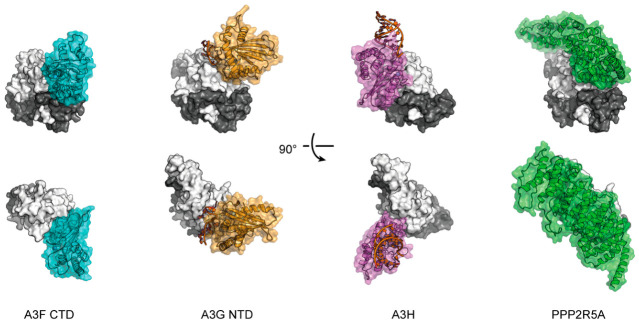
Complex structures of Vif in complex with A3 family proteins and PPP2R5A. The cryo-EM structures of Vif in complex with A3F CTD (PDB 6NIL), A3G (PDB 8CX0), A3H (PDB 8FVI), and PPP2R5A (PDB 8SZK) were generated by PyMOL v2. The structural data for the A3G CTD in the cryo-EM structure (PDB 8CX0) are omitted for simplicity. Vif and CBF-β are shown with light gray and dark gray surfaces, respectively, whereas A3F CTD, A3G NTD, A3H, and PPP2R5A are displayed with ribbons and semitransparent surfaces colored in cyan, light orange, violet, and light green, respectively. RNA and a zinc ion are represented with an orange ribbon and an aqua sphere, respectively.

Comparison of a series of cryo-EM structures of Vif with A3F CTD, A3G, and A3H revealed their distinct binding modes (Figure 4). The three distinct, nonoverlapping regions of Vif separately recognize A3F CTD, A3G, and A3H. Furthermore, their binding orientations and interface sizes (A3G > A3F CTD > A3H) are variable [23,24,27,28].

In contrast, the three sets of A3-Vif interfaces appear to share similar electrostatic characteristics, despite their variable charge distributions (Figure 2B and Figure 3B). The protein–protein interfaces on each A3 tend to be negatively charged, while their counterparts on Vif are more likely to be positively charged. Notably, in the case of the A3G-Vif complex, a negatively charged ssRNA molecule is accommodated between positively charged interfaces on A3G and Vif. Electrostatic complementarity at the interfaces of each complex may facilitate favorable intermolecular interactions, including not only hydrophobic contacts but also salt bridges and hydrogen bonds [23,24,25,26,27,28]. Therefore, this kind of complementarity may drive sufficient stability and specificity for the binding of Vif to each A3 [60,61]. This notion is further supported by the findings that charge-swapping mutations on A3 interfaces, such as A3G D128K and A3F E324K, confer resistance to HIV-1 Vif-mediated degradation [40,62]. Hence, the HIV-1 *vif* gene may have evolved to acquire three relatively positively charged surface areas to independently recognize relatively negatively charged surfaces on human A3F, human A3G-ssRNA, and human A3H. Given that the overall charge of the Vif protein is highly positive [22], there could be additional reasons for the presence of these positively charged surfaces on Vif, such as increasing accessibility to A3 proteins bound to highly negatively charged nucleic acids in cells.

## 7. PPP2R5A

Finally, we briefly describe the structural mechanism underlying the Vif-mediated downregulation of the function of another host factor, the Protein Phosphatase 2 Regulatory Subunit B’Alpha (PPP2R5A), which induces G2/M cell cycle arrest [35,63]. A recent cryo-EM study revealed the complex structure of PPP2R5A with HIV-1 Vif [64] (PDB 8SZK) (Figure 4). The interface partially overlaps with those for A3G, A3F, and A3H hapII and is slightly larger than that for A3G-ssRNA [20,63,64]. However, the conservation of the Vif amino acid residues required for PPP2R5A interactions varies by region and subtype [35,65]. Hence, the downregulation of PPP2R5A might be an auxiliary Vif function for HIV-1 production [66].

## 8. Summary

In this review, we summarize the current knowledge concerning the structural features of multifaceted Vif interactions with human A3 family proteins. This information will be valuable for developing a new class of anti-HIV-1 drugs that interfere with interactions at A3-Vif interfaces. Although the Vif-binding pockets of A3s appear shallow for the design of high-affinity small-molecule inhibitors, multiple groups have nevertheless identified candidate inhibitors targeting the Vif-A3 interaction [67]. In addition, it will also aid in understanding the evolutionary conflict between the HIV-1/SIV lineage and their hosts. Amino acids are not fully conserved at the corresponding interfaces of primate A3 family proteins and HIV-1/SIVs. Hence, as proposed by the “wobble model” [68], the three types of interfaces between HIV-1 Vif and human A3s might be the consequences of three independent pathways for the adaptation of ancestral Vif-A3 interactions. However, the detailed mechanism behind these evolutionary dynamics remains unclear. Further studies are needed to understand the evolutionary conflict between primate lentiviral Vif and host A3s.

## Data Availability

No new data were created or analyzed in this study. Data sharing is not applicable to this article.

## Abbreviations

The following abbreviations are used in this manuscript:

A3	APOBEC3
A3A	APOBEC3A
A3B	APOBEC3B
A3C	APOBEC3C
A3D	APOBEC3D
A3F	APOBEC3F
A3G	APOBEC3G
A3H	APOBEC3H
APOBEC3	Apolipoprotein B mRNA editing enzyme catalytic polypeptide 3
CBF-β	Core-binding factor subunit beta
cpzA3H	chimpanzee A3H
cryo-EM	cryogenic electron microscopy
CTD	C-terminal domain
CUL5	Cullin-5
dsRNA	double-stranded RNA
ELOB	Elongin B
ELOC	Elongin C
hapI	haplotype I
hapII	haplotype II
HIV-1	Human immunodeficiency virus type 1
HUWE1	HECT, UBA and WWE domain-containing E3 ubiquitin protein ligase 1
NTD	N-terminal domain
PDB	Protein Data Bank
PPP2R5A	Protein phosphatase 2 regulatory subunit B’alpha
RBX2	RING-box protein 2
RMSD	Root-mean-square deviation
SIV	Simian immunodeficiency virus
ssRNA	single-stranded RNA
UBR4	Ubiquitin protein ligase E3 component N-recognin 4
UBR5	Ubiquitin protein ligase E3 component N-recognin 5
Vif	Viral infectivity factor
